# Lung Ultrasound Examination in Patients with SARS-CoV-2 Infection: Multicenter Study

**DOI:** 10.3390/jcm10153255

**Published:** 2021-07-23

**Authors:** Natalia Buda, Jolanta Cylwik, Katarzyna Mróz, Renata Rudzińska, Paweł Dubik, Agnieszka Malczewska, Aleksandra Oraczewska, Szymon Skoczyński, Anna Suska, Tomasz Górecki, Konrad Mendrala, Jakub Piotrkowski, Wojciech Gola, Elena Segura-Grau, Anna Zamojska, Marcin Wełnicki

**Affiliations:** 1 Department of Internal Medicine, Connective Tissue Diseases and Geriatrics, Medical University of Gdansk, 80-210 Gdansk, Poland; natabud@wp.pl; 2 Anesthesiology and Intensive Care Unit, Mazovia Regional Hospital in Siedlce, 08-110 Siedlce, Poland; jolacylwik@o2.pl; 3 Department of Internal Medicine and Diabetology, Kielce Hospital of St. Aleksandra LLC, 25-316 Kielce, Poland; kat.jurczyga@gmail.com; 4 Clinical Department of Anesthesiology and Intensive Care, Central Clinical Hospital of the Ministry of Internal Affairs and Administration in Warsaw, 02-507 Warsaw, Poland; renata.kwiecinska@gmail.com; 5 Department of Anesthesiology and Intensive Therapy, Healthcare Center of the Ministry of Internal Affairs and Administration Named after Sergeant Grzegorz Załoga in Katowice, 40-052 Katowice, Poland; paweldubik@wp.pl; 6 Anesthesiology and Intensive Care Unit, Polish Red Cross Maritime Hospital in Gdynia, 81-621 Gdynia, Poland; malczewskaaga@wp.pl; 7 Department of Pneumonology, School of Medicine in Katowice, Medical University of Silesia in Katowice, 40-055 Katowice, Poland; oraczewskaaleksandra@gmail.com (A.O.); simon.mds@poczta.fm (S.S.); 8 Emergency Department, The University Hospital in Cracow, Jagiellonian University Medical College, 31-008 Cracow, Poland; an.suska@gmail.com; 9 Department of Medical Education, Faculty of Medicine, Jagiellonian University Medical College, 31-008 Cracow, Poland; t.gorecki@uj.edu.pl; 10 Department of Cardiac Anesthesiology and Intensive Therapy, Medical University of Silesia, 40-055 Katowice, Poland; k.mendrala@gmail.com; 11 Department of Internal Medicine, Gastroenterology and Oncological Cardiology, Independent Public Health Care Facility of the Ministry of Internal Affairs and Administration with the Oncology Center in Olsztyn, 10-228 Olsztyn, Poland; kuba.piotrkowski@gmail.com; 12 Faculty of Medicine and Health Sciences, Jan Kochanowski University, 25-369 Kielce, Poland; golawojtek@gmail.com; 13 Anesthesiology Department, Tondela-Viseu Hospital Center, EPE, 3504-509 Viseu, Portugal; elenasegura12@hotmail.com; 14 Department of Econometrics, University of Gdansk, 80-210 Gdansk, Poland; a.zamojska7@gmail.com; 15 3rd Department of Internal Medicine and Cardiology, Medical University of Warsaw, 04-749 Warsaw, Poland

**Keywords:** LUS, COVID-19, interstitial pneumonia, chest sonography, pneumonia

## Abstract

Background: The COVID-19 pandemic has, by necessity, contributed to rapid advancements in medicine. Owing to the necessity of following strict anti-epidemic sanitary measures when taking care of infected patients, the accessibility of standard diagnostic methods may be limited. Consequently, the significance and potential of bedside diagnostic modalities increase, including lung ultrasound (LUS). Method: Multicenter registry study involving adult patients with confirmed COVID-19, for whom LUS was performed. Results: A total of 228 patients (61% males) qualified for the study. The average age was 60 years (±14), 40% were older than 65 years of age. In 130 from 173 hospitalized patients, HRCT (high-resolution computed tomography) was performed. In 80% of patients, LUS findings indicated interstitial pneumonia. In hospitalized patients multifocally located single B-lines, symmetrical B-lines, and areas of white lung were significantly more frequent as compared to ambulatory patients. LUS findings, both those indicating interstitial syndrome and consolidations, were positively correlated with HRCT images. As compared to HRCT, the sensitivity and specificity of LUS in detecting interstitial pneumonia were 97% and 100%, respectively. Conclusions: As compared to HRCT, LUS is characterized by a very high sensitivity and specificity in detecting interstitial pneumonia in COVID-19 patients. Potentially, LUS can be a particularly useful diagnostic modality for COVID-19 patients pneumonia.

## 1. Introduction

Bilateral lung lesions are found in as many as 75% of patients with SARS-CoV-2 in a chest computed tomography (CT) scan [1]. In over 60% of patients, the oxygenation index decreases and as many as 20% of hospitalized patients develop respiratory failure or acute respiratory distress syndrome (ARDS) [1]. However, the most frequent symptoms of SARS-CoV-2 infection, apart from fever (approx. 80%), include cough (approx. 63%) and fatigue (46%). Variously described sensations of shortness of breath or difficulties with breathing are reported by every third patient. Since the onset of the pandemic, clinicians have also reported ‘silent hypoxemia’ in COVID-19 patients [2]. Hence, the actual extent of COVID-19-related lung involvement may be underestimated. In this group of patients, high-resolution computed tomography (HRCT) is the primary diagnostic imaging tool for lung assessment. HRCT is the gold standard in diagnostic imaging of pulmonary interstitial involvement; however, it requires a patient to be transported to the radiology unit and is also associated with exposure to ionizing radiation. These disadvantages, as well as the patient’s often dynamically changing clinical status, justify searching for noninvasive methods, alternative or complementary to HRCT, facilitating multiple lung evaluations in hospitalized COVID-19 patients. Lung ultrasound (LUS) appears to be a particularly promising tool in this respect. The significance of this modality in detecting interstitial pneumonia of various etiologies has increased recently, and since the onset of the COVID-19 pandemic, LUS has been more often employed for the evaluation of lung involvement also in this group of patients [3,4,5]. Considering the often dynamic nature of hospitalization of a patient with COVID-19, it can be assumed that LUS in many cases could be a valuable alternative to HRCT. Therefore, the main aim of our study was to compare LUS images with the gold standard—that is, HRCT. In addition, we wanted to compare the outpatient LUS results with the inpatient studies.

## 2. Materials and Methods

### 2.1. Ethics Committee

The study was approved by the Ethics Committee of the Medical University of Gdańsk (NKBBN/647/2020).

### 2.2. Patient Qualification

The inclusion criterion was SARS-CoV-2 infection confirmed by an antigen test and/or a positive RT-PCR test. Patients younger than 18 years of age and patients without confirmed SARS-CoV-2 (negative antigen test and/or negative SARS-CoV-2 RT-PCR test) were excluded. All patients consented to the participation in the study and the processing and anonymized use of images and/or recorded video material (cine loops) of performed imaging examinations.

### 2.3. Research Team

This study was a multicenter register involving 13 centers from all over Poland and was conducted by an interdisciplinary team of physicians who specialize in various fields of medicine (internal medicine, pulmonology, anesthesiology and intensive care, emergency medicine, and infectious diseases) and paramedics. All team members have long-standing practical experience in employing LUS as a diagnostic tool for patients in their respective medical specialties (ranging from 2 to 11 years), ensuring the realization of study objectives.

### 2.4. Study Protocol

Indications for LUS and chest tomography were established by research team members as part of their routine, everyday diagnostic activities.

LUS was performed for all patients qualified for the study. The examinations were conducted with various ultrasound devices available in particular centers. For lung evaluation, both convex and linear transducers were employed (depending on the ultrasound machine in a given center). In order to optimize lung imaging, the examination was conducted with ‘cosmetic’ filters switched off (e.g., speckle reduction, compound imaging, tissue harmonic imaging). In cases of additional indications, Color Doppler was also used (evaluation of flows in consolidations). The right and left zones of the chest were examined separately. The transducer was placed longitudinally over the intercostal space to evaluate the largest possible area of the pleura. If the patient’s condition allowed, the examination was performed in a sitting position and the entire surface of the chest was assessed. In more severe cases, the anterolateral surfaces of the thorax in patients in the supine position and the posterolateral surfaces in patients in the prone position were assessed. Obtained images were recorded in the device internal memory or on electronic data storage devices. Ultrasound findings included:

(1) Abnormalities within the pleural line (thickened, irregular, fragmented);
(2) Artifacts: multifocally located single B-line artifacts, multifocal multiple (confluent) B-line artifacts, bilateral and symmetrical B-lines, white lung, spared areas;
(3) Consolidations: small subpleural consolidations (up to 5 mm) accompanied with C-line artifacts, large consolidations involving segments and/or lobes, pleural effusion.

HRCT examinations were performed following the standard protocol in a given center. Laboratory test results were interpreted in accordance with reference values in a given laboratory.

### 2.5. Database

All data were anonymized. Research team members input the following data into an electronic form created for the purpose of the study: age, sex, medical COVID-19-related history (including the day when first symptoms appeared) and chronic comorbidities. Additionally, the form contained a detailed evaluation of LUS findings determined by the operator and description of the chest CT. The filled in form and obtained anonymized LUS recordings (cine loops or pictures) as well as CT images were sent to the team supervising the creation of the integrated database. At this stage, ultrasound images were reevaluated by the most experienced members of the research team and blindly compared with CT images.

### 2.6. Statistical Analysis

At the first stage of the study, the analysis of statistical series characteristics was performed (Data Generating Process). The classical descriptive statistics were employed for continuous variables (mean and standard deviation). For nominal variables, respective fractions were compared. At the second stage, two tests were used for drawing statistical conclusions: a Z-test and a chi-square test. The Z-test was chosen to compare the significance of fraction differences between groups, and the chi-square test to determine the compatibility of characteristics referring to the cooccurrence of symptoms in the studied patients. For both tests, *p*-value = 0.05 was regarded as statistically significant. STATA 16 software was used for calculations.

## 3. Results

### 3.1. Study Group

In total, 228 patients qualified for the study, 61% (139) of the group were males and 39% (89) were females. The average age was 60 years (±14), patients older than 65 years constituted 40% (93) of the group.

A positive antigen test result was obtained for 14 patients. The remaining 214 patients had a positive SARS-CoV-2 RT-PCR result based on the nasopharyngeal swab; in 198 patients as the first test performed, in 8 patients as the second test, and in 2 patients as the third test. Six patients had a positive SARS-CoV-2 RT-PCR result based on the examination of the washings obtained during bronchofiberoscopy.

The most frequent comorbidities included: obesity (52%), congestive heart failure (42%), and diabetes (33%). Asthma or chronic obstructive pulmonary disease (COPD) were found in 8.2%, and interstitial lung disease only in 1.5% of patients. More than one comorbidity was detected in two-thirds of the study group, and more than two coexisting diseases were found in 89 patients (66%).

Ambulatory patients constituted 24% (55 individuals) of the study group, the remaining patients were hospitalized. Among 173 hospitalized patients, 87% (117) required passive oxygen therapy, and respiratory failure occurred in 59% (134), with nearly half of that group (48%, 64) requiring mechanical ventilation. Multiple-organ failure developed in 32% of hospitalized patients (56), each of them requiring infusions of pressor amines, and seven needed renal replacement therapy. Finally, almost half (46%) of patients hospitalized due to SARS-CoV-2 infection required intensive care.

In the study group, 160 patients received the following medication: antiviral drugs—31 patients (19.3%); COVID-19 convalescent plasma transfusion—54 (33.8%); tocilizumab—12 (7.5%); dexamethasone—86 (53.7%); low molecular weight heparin in prophylactic or therapeutic doses—108 patients (83%). None of the aforementioned medications were administered in 13 (7.5%) hospitalized patients.

### 3.2. Imaging Examinations (LUS and HRCT)

LUS was performed for 55 ambulatory patients, 93 patients treated in departments of internal medicine, and 80 patients in intensive care units (ICU) dedicated to COVID-19 patients. HRCT was performed for 130 hospitalized patients, in 74% (96) of these patients HRCT was conducted within 4 days before LUS.

In the case of 80% of patients (183), LUS findings indicated interstitial pneumonia. The visualized changes included:

- Abnormalities within the pleural line (irregular, fragmented);
- Multifocally located single B-line artifacts;
- Multifocal multiple (confluent) B-line artifacts;
- Bilateral and symmetrical B-lines;
- White lung;
- Spared areas;
- Small subpleural consolidations (up to 5 mm) accompanied with C-line artifacts;
- Large consolidations involving segments and/or lobes;
- Pleural effusions.

**Figure 1:**
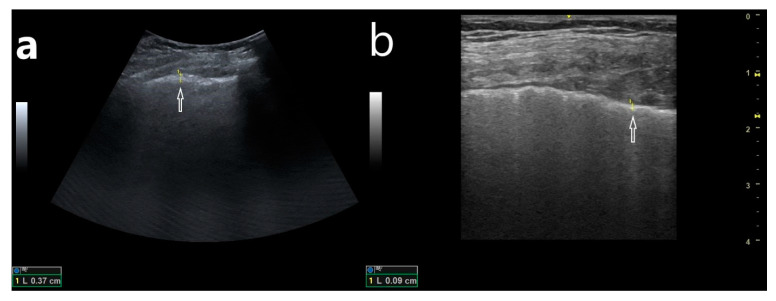
(**a**) B-line artifacts, arising from an apparently thickened pleural line when it was evaluated with a convex transducer (3.1 mm); (**b**) evaluation of the pleural line in the same localization, the pleural line of normal thickness, assessed with a linear transducer (0.09 mm).

**Figure 2:**
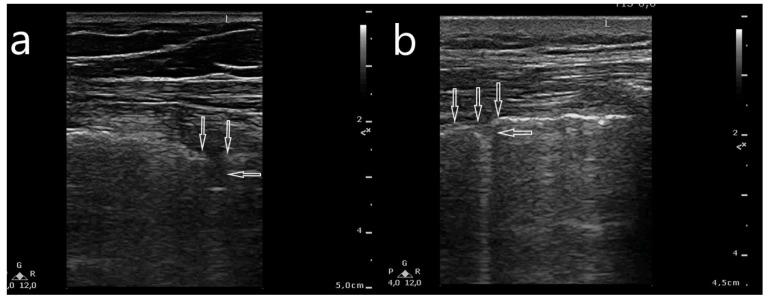
Abnormalities within the pleural line evaluated with a linear transducer: (**a**) fragmented pleural line corresponding to its segmental hypoechogenicity (between vertical arrows) and a small subpleural consolidation (horizontal arrow) (<5 mm); (**b**) irregular pleural line (vertical arrows), and a small subpleural consolidation (horizontal arrow).

**Figure 3:**
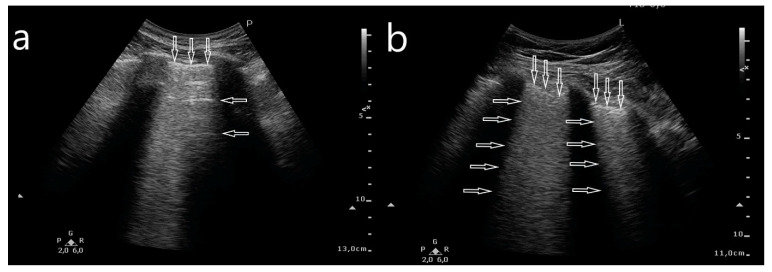
B-line artifacts evaluated with a convex transducer: (**a**) A-line artifacts (horizontal arrows) and pleural line (vertical arrow). (**b**) White lung: pleural line (vertical arrow) and confluent B-line artifacts (horizontal arrows).

**Figure 4:**
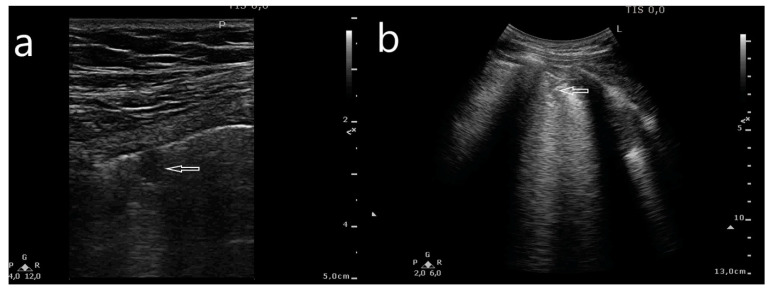
Subpleural consolidation, oval (**a**) and wedge-shaped (**b**), with a C-line artifact arising from the bottom margin of the subpleural consolidation (horizontal arrows).

**Figure 5:**
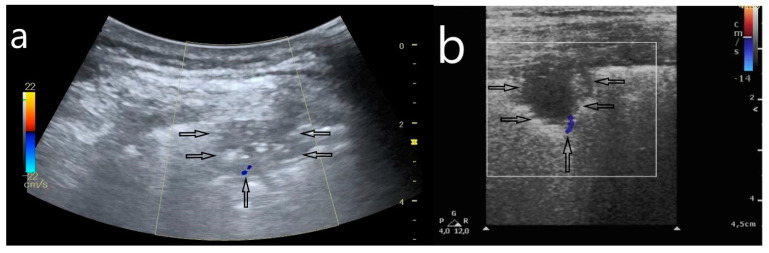
Subpleural consolidation due to pulmonary embolism secondary to COVID-19. (**a**,**b**) Wedge-shaped subpleural consolidation (horizontal arrows), with a ‘vascular sign’—flow amputation visible in Color Doppler (vertical arrow).

A thickened pleural line was not visualized in the study group. Figure 1, Figure 2, Figure 3, Figure 4 and Figure 5.

**Figure 6:**
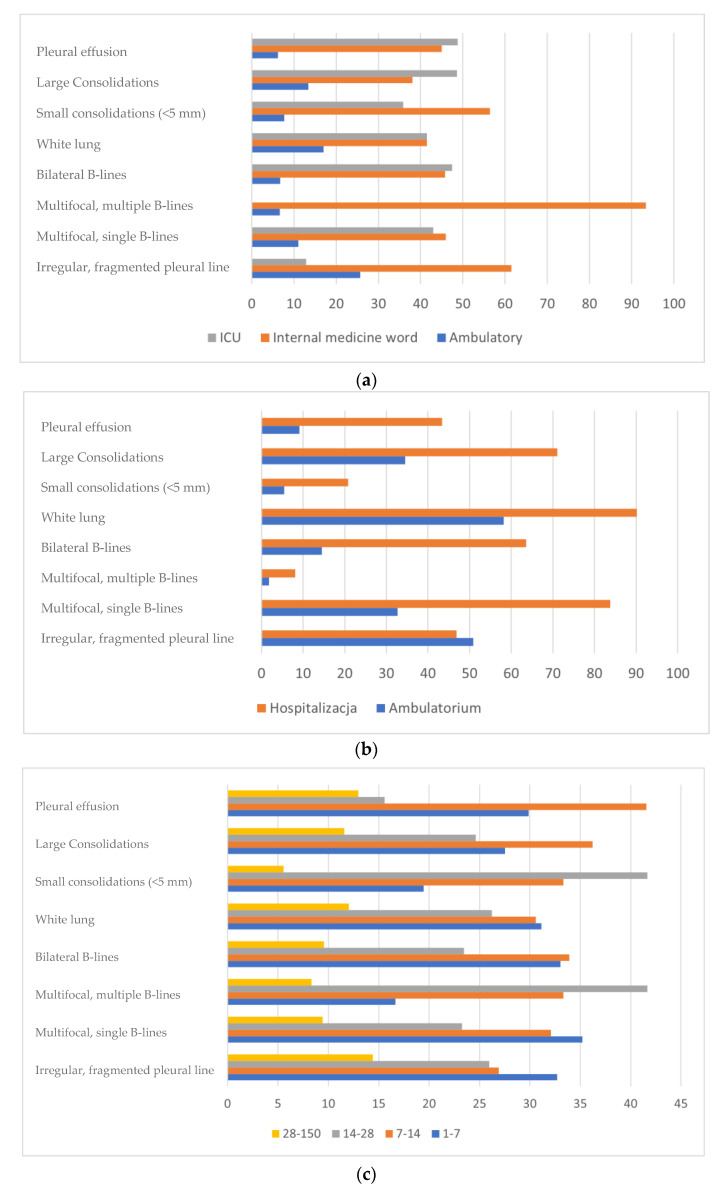
Figures demonstrate the presence of specific LUS findings in patients with the SARS-CoV-2 infection depending on: (**a**) place of treatment (ambulatory, department of internal medicine, intensive care unit), (**b**) treatment mode (ambulatory and hospitalization), (**c**) period of illness.

As shown in Table 1 and Table 2, multifocal single B-lines and symmetrical B-lines with lung and large consolidations were significantly more frequent in hospitalized patients. Those differences are also shown in Figure 6a,b. LUS results also depended on the time between the onset of the disease and the date of the investigation (shown in Table 3 and Figure 6c). As the main aim of our study was to compare LUS images with HRCT, a statistical analysis was also applied to compare findings in both image modalities. A positive correlation was revealed between the following features:

(a) Ground glass opacity in an HRCT scan and white lung sign in an LUS image (*p* = 0.04).
(b) Small subpleural consolidations in an HRCT scan and small subpleural consolidations in an LUS image (*p* = 0.006), and single but multifocal B-line artifacts (*p* = 0.001).
(c) Large consolidations involving segments or lobes in an HRCT scan and large consolidations in an LUS image (*p* = 0.007).
(d) Multifocal abnormalities in an HRCT scan and multifocal abnormalities in an LUS image (*p* = 0.002).

Ultrasound findings indicating interstitial pneumonia correlated with the respective HRCT images, with a sensitivity of 97%, and a specificity of 100%, PPV 1, NPV 0.5.

## 4. Discussion

SARS-CoV-2-related pneumonia is of an interstitial nature. Available diagnostic imaging methods to detect pulmonary interstitial involvement include X-ray and, presently more frequently, LUS and HRCT which remain the gold standard.

Chest radiographs in COVID-19 patients depicted band or reticular changes and multifocal changes of a ground glass pattern. Sometimes, features of subsegmental atelectasis may be present, caused by a small caliber airways obstruction [6]. Abnormalities were primarily localized in the middle and lower fields, mostly peripherally. The sensitivity of radiography in detecting COVID-19-related interstitial involvement differs, depending on the period of illness. In one of the multicenter studies, Stephanie et al. determined the sensitivity of chest X-ray to be nearly 55% if the radiograph was taken up to the 2nd day following first symptoms. After 11 days following the occurrence of first symptoms, the sensitivity of X-ray increases to 79%. With the progression of the illness, the specificity of this examination decreases from 83% to 70% [7]. However, it is also possible that chest radiography will not detect pulmonary interstitial involvement. Consequently, owing to a relatively low sensitivity, chest X-ray is not a preferred method of a radiological assessment of COVID-19 patients, yet can be significant in the differential diagnosis of lung diseases.

As it was mentioned above, HRCT remains the gold standard. Findings typical of COVID-19 detected in this examination included a ground glass opacity (83%), ground glass opacity with mixed consolidations (58%), interlobular septal thickening (48%), and air bronchograms (46%). Less frequent signs included a crazy paving pattern, pleural effusion, bronchiectasis, pericardial effusion, and lymphadenopathy [8]. A meta-analysis of six studies of 1431 COVID-19 cases in total, published by Hug J. A. Adams, proves a very high sensitivity of chest CT (92.9–97%), with a simultaneous large range of its specificity (25–71.9%) [9]. Although HRCT is undoubtedly the gold standard in detecting interstitial pneumonia, this examination has some disadvantages. It requires the patient to be exposed to ionizing radiation, which limits the possibility of reexamination. Apart from the basic limitation of HRCT during the pandemic and strict sanitary measures, it is necessary to undertake the patient’s intrahospital transport. Moreover, the patient’s clinical status, if serious, also impacts the decision whether to perform CT.

During the pandemic, numerous publications dealing with the application of LUS in COVID-19 have appeared. Data from the reports indicate that abnormalities are visible in LUS earlier as compared to chest X-ray. The sensitivity of detecting interstitial lung involvement in one study was 94.1%, and specificity was 84.4% [10]. Interstitial lung involvement due to viral infections detected in LUS was reported during the AH1N1 flu epidemic. These were, however, single reports concerning a small number of patients, mostly a pediatric population. Presently, the majority of publications reporting the application of LUS in COVID-19 diagnostics are case studies, original papers concerning small groups of patients or review papers. Pulmonary COVID-19-related findings in LUS described in literature include abnormalities within the pleural line (irregular, fragmented, thickened), vertical artifacts, and consolidations. In our study, we did not observe a thickened pleural line in any of the examined patients. We believe that this is related to the methodological assumptions of the study. The pleural line should be assessed with a linear transducer. If only a convex transducer is employed, the pleural line may seem to be thickened. The evaluation with the linear transducer does not confirm the thickening.

Another group of COVID-19-related findings in LUS are vertical artifacts. Each publication devoted to this subject reports the presence of B-line artifacts. B-lines may occur as single artifacts or as confluent artifacts creating the white lung sign (termed by some authors the waterfall sign). We described the presence of C-line artifacts arising from the bottom margin of subpleural consolidations. In the majority of consolidations, the signal was hyper intensified below their bottom margin, which is termed as a C-line artifact. Unfortunately, in the literature, C-line artifacts are often taken for B-line artifacts that, by definition, arise from the pleural line and not from the bottom margin of subpleural lesions. Additionally, B-line artifacts, being vertical and laser-like, reach the bottom edge of the screen.

The third type of LUS findings in interstitial pneumonia are consolidations, mostly small subpleural lesions. In hospitalized patients, however, also large consolidations are detected, corresponding to inflammatory lesions. It should be remembered that in the course of COVID-19, patients often develop thromboembolic complications. Subpleural consolidations may then correspond to peripheral myocardial changes due to pulmonary embolism.

In most publications devoted to the application of LUS in COVID-19 the examination was conducted with a convex transducer or another low frequency transducer (microconvex or phase-array). A linear transducer was used only in single studies. We believe that the choice of transducers for the evaluation of patients with interstitial pneumonia is decisive for the described LUS findings. Consequently, our study protocol assumed the application of convex and linear transducers as often as possible.

The authors of the reports also suggest various techniques for the visualization of pulmonary lesions. Some are based on the BLUE or extended BLUE protocol. Others, due to multifocal lesions, suggest scanning the entire chest surface or evaluation in 10–12 lung zones [11,12,13].

Calvo-Cebrian et al. demonstrated that the advancement of pulmonary involvement visualized in LUS correlated with the increased risk of hospitalization [14]. Others indicated the relationship between the severity of pathologies detected in LUS and the risk of intubation [15]. The results of our study regarding the differences between the changes found in outpatients and inpatients were consistent with these observations. Ultrasound evaluation appears to be a valuable tool for the lung assessment in COVID-19 patients, and its sensitivity for detecting pulmonary interstitial inflammatory involvement is higher that of chest X-ray [16].

In our study, comparing the LUS and HRCT results, the sensitivity and specificity for detection of abnormalities typical of interstitial pneumonia was very high, reaching 97% and 100%, respectively. These values appear significantly higher than in the studies conducted before the COVID-19 era [10].

We observed a statistically significant correlation between LUS images and HRCT scans as regards to the multifocality of changes and the presence of large abnormalities (in LUS large consolidations). Moreover, from a clinical standpoint, the correlation between the white lung sign in LUS images and ground glass opacities in HRCT scans is significant. We also confirmed the compatibility of both examinations as regards to multifocality of lesion localization.

The results of our study, together with the results of other researchers, emphasize the important role of LUS in the diagnosis of pneumonia in COVID-19. It should be stressed that in recent reports from Volpicelli et al., the most important multicenter study in the field of lung ultrasound, it has been evidenced that the presence of interstitial lung involvement detected in LUS may indicate COVID-19 in patients who did not have an RT-PCR test, with a sensitivity depending on the phenotype of clinical symptoms, but exceeding 80% [17]. Senter et al. revealed some very interesting correlations between LUS images and the results of laboratory tests and reported a significant correlation between the CRP level and the presence of subpleural consolidations as well as a reverse correlation between the lymphocyte count and the intensification of interstitial involvement presented as B-lines [18].

Considering the above, it appears justifiable to suggest LUS as a diagnostic tool alternative to HRCT, one than can provide important information concerning the extent of COVID-19-related lung involvement. This conclusion is confirmed by the comments of other authors [19]. It is also worth paying attention to the studies by Biasucci et al., who prove the possibility of determining the risk of the necessity to use mechanical ventilation and non-invasive ventilation (NIVM) failure based on the LUS results [20]. On the other hand, Bonadia et al. prove that the LUS results conducted in the emergency room allow not only to determine the risk of hospitalization in the intensive care unit, but also correlate with the patient’s risk of death [21]. Taking it all into consideration, it can be stated that LUS is helpful for making crucial therapeutic decisions during the pandemic—the necessity of hospitalization, qualification for the type of respiratory support (passive oxygen therapy, AIRVO or mechanical ventilation), as well as during pharmacotherapy (indications for antiviral treatment or possible administration of antibiotics, or even increasing a low molecular weight heparin dose when peripheral embolism is suspected).

### Limitations

The main limitation of our study was the impossibility of performing both LUS and HRCT in the entire study group. Due to the epidemic conditions, the serious general clinical status of some patients, treatment in ICUs and the impossibility of transporting clinically unstable patients to the radiology unit, as well as conservative ambulatory treatment of respiratory system-stable patients, a large number of patients could not undergo a chest tomography. The comparative analysis (HRCT vs. LUS) was carried out only for those patients who had both examinations performed within a period not exceeding 4 days.

Another limitation is the difference in the number of lung fields scanned. Cooperating patients, of good or average general clinical status, had the entire lung surfaces scanned, including the anterior, lateral and posterior chest wall. However, in ICU patients, LUS was mostly performed over the anterior and posterolateral chest wall, excluding paraspinal sections. The objective of the study was to scan the largest possible lung surface.

It should be also remembered that, before the pandemic, the detection of pulmonary interstitial involvement in any imaging modality initiated differential diagnosis. Presently, considering the epidemic situation and clinical compatibility, COVID-19 is the most frequent cause of such involvement. However, in the commentary on the results of research on the use of LUS in COVID-19, it is rightly noted that the morphology of the lesions does not clearly indicate a specific pathogen [22].

## 5. Conclusions

1. LUS is an efficient imaging modality for the evaluation of interstitial lung involvement and is sensitively and specifically highly comparable to that of HRCT.
2. Sonomorphology of pulmonary abnormalities depends on the time elapsed sinceSARS-CoV-2 infection. The detection of multifocal interstitial involvement in ambulatory conditions may indicate the necessity for the hospitalization of infected patients.

## Figures and Tables

**Table 1:** Incidence of specific LUS findings, depending on the patient group: 1—ambulatory; 2—department of internal medicine; 3—intensive care unit.

Place of Treatment	Irregular, Fragmented Pleural Line	Multifocal, Single B-Lines	Multifocal, Multiple B-Lines	Symmetrical B-Lines	White Lung	Small Consolidations (<5 mm)	Large Consolidations	Pleural Effusion
1	26	11	7	7	17	8	13	6
2	61	46	93	46	41	56	38	45
3	13	43	0	47	41	36	49	49

The background colour, from green through yellow and orange to red, symbolizes the increasing frequency of specific LUS findings.

**Table 2:** Incidence of specific LUS findings in ambulatory and hospitalized patients. 1—ambulatory; 2—department of internal medicine; 3—intensive care unit.

Place of Treatment	Irregular, Fragmented Pleural Line	Multifocal, Single B-Lines	Multifocal, Multiple B-Lines	Symmetrical B-Lines	White Lung	Small Consolidations (<5 mm)	Large Consolidations	Pleural Effusion
1	51	33	2	15	58	5	35	9
2 + 3	47	84	8	64	90	21	71	43
Fraction difference test 1 and 2 together with 3	0.365	−4.957	−0.219	−3.608	−4.563	−0.669	−3.087	−1.497
*p*-value	0.715	0.000	0.827	0.000	0.000	0.504	0.002	0.134

The background colour, from green through yellow and orange to red, symbolizes the increasing frequency of specific LUS findings. Significant difference (*p*-value) is marked in red.

**Table 3:** Summarized incidence of specific LUS findings depending on the period of illness.

Period of Illness in Days	Irregular, Fragmented Pleural Line	Multifocal, Single B-Lines	Multifocal, Multiple B-Lines	Symmetrical B-Lines	White Lung	Small Consolidations (<5 mm)	Large Consolidations	Pleural Effusion
1–7	33	35	17	33	31	19	28	30
7–14	27	32	33	34	31	33	36	42
14–28	26	23	42	23	26	42	25	16
28–150	14	9	8	10	12	6	12	13

The background color, from green through yellow and orange to red, symbolizes the increasing frequency of specific LUS findings.

## Data Availability

Data are contained within the article.
